# Recent incidence trend of elderly patients with glioblastoma in the United States, 2000–2017

**DOI:** 10.1186/s12885-020-07778-1

**Published:** 2021-01-12

**Authors:** Boran Chen, Chaoyue Chen, Yang Zhang, Jianguo Xu

**Affiliations:** 1 Department of Neurosurgery, West China Hospital, Sichuan University, West China Hosptial, No. 37, GuoXue Alley, Chengdu, 610041 People’s Republic of China; 2 West China School of Medicine, Sichuan University, Chengdu, China; 3 State Key Laboratory of Biotherapy and Cancer Center, West China Hospital, Sichuan University, and Collaborative Innovation Center for Biotherapy, Chengdu, China

**Keywords:** Glioblastoma, Elderly, Age-adjusted incidence rate, Annual percentage change, SEER, Join-point analysis, Age-period-cohort analysis

## Abstract

**Background:**

The incidence of glioblastoma increases significantly with age. With the growing and aging population, there is a lack of comprehensive analysis of recent glioblastoma incidence trend in the United States. This study aims to provide in-depth description of the patterns of incidence trends and to examine the age-period-cohort effects to the trends of glioblastoma specific to elderly patients.

**Methods:**

The incidence rates were age-adjusted and reported per 100,000 population. We calculated the annual percent change (APC) in incidence using the Joinpoint Regression Program and conducted an age-period-cohort analysis of elderly glioblastoma reported between 2000 and 2017 to the Surveillance Epidemiology and End Results (SEER) 18 registry database.

**Results:**

The overall incidence rate of elderly patients with glioblastoma was 13.16 per 100,000 (95% CI, 12.99–13.32) from 2000 to 2017. Non-Hispanic whites (20,406, 83.6%) made up the majority. The incidence rate of male was about 1.62 times that of female. The trend of incidence remained stable and there was a non-significant increasing tendency for all elderly patients (APC 0.3, 95% CI, − 0.1 to 0.7, *p* = 0.111). There was a significantly increasing incidence trend for non-Hispanic white (APC 0.6, 95% CI, 0.2 to 1.1, *p* = 0.013), supratentorial location (APC 0.7, 95% CI, 0.2 to 1.3, *p* = 0.016), tumor size < 4 cm (APC 2.5, 95% CI, 1.4 to 3.6, *p* < 0.001), and a significantly decreasing trend for overlapping/NOS location (APC -0.9, 95% CI, − 1.6 to − 0.2, *p* = 0.012), and unknown tumor size (APC -4.9, 95% CI, − 6.6 to − 3.3, *p* < 0.001). The age-period-cohort analysis showed the effect of age on incidence trends (*p*< 0.001, Wald test), while did not indicate the period and cohort effects of the incidence trends of glioblastoma (*p* = 0.063 and *p* =0.536, respectively, Wald test).

**Conclusion:**

The overall incidence of glioblastoma in the elderly population remained stable between 2000 and 2017. Period and cohort effects were not evident in the trend of glioblastoma incidence. Future population-based studies exploring the difference in the trend of glioblastoma incidence by specific molecular subgroups are warranted to further our understanding of the etiology of glioblastoma.

## Background

Glioblastoma is the most common and aggressive primary central nervous system tumor with an age-adjusted incidence rate of 3.22 per 100,000 population, which accounts for 57.3% of all gliomas and 48.3% of all malignant brain tumors [1]. The incidence of glioblastoma increases significantly with age and the median age at diagnosis is 65 years [1, 2]. Patients with glioblastoma have constantly poor prognosis over the past decades with a median survival of less than 2 years, despite therapeutic advances [3, 4]. The landmark randomized controlled trial presented by Stupp et al. suggested that patients with glioblastoma may benefit from radiotherapy with concomitant temozolomide followed by six cycles of temozolomide [4]. However, this trial only did not include elderly patients. Advanced age is a well-established negative prognostic factor [5, 6]. The median survival is less than 10 months among elderly patients with short-course radiotherapy plus temozolomide, according to a phase 3 randomized controlled trial [7]. Elderly patients with glioblastoma have worse prognosis and are therefore an important subgroup. However, limited studies have focused on the incidence trend of elderly glioblastoma. Dobes et al. reported an increasing incidence of glioblastoma in patients aged over 65 years from 2000 to 2008 in Australia with an annual percentage change (APC) of 3.0 (95% confidence interval [CI], 0.5–5.6) [8]. Korja et al. reported increased proportion (from 24 to 27%) of patients aged over 70 years with glioblastoma between 2000 and 2013 in Finland [9]. The Central Brain Tumor Registry of the US (CBTRUS) report 2019 provided incidence rates and incidence trends of glioblastoma by age group (0–14 years, 15–39 years, and 40+ years) [1]. It exists a vacancy in the incidence trend of patients with glioblastoma aged 65 years or older in CBTRUS report 2019. Hence there is a lack of comprehensive analysis of recent incidence trend of elderly patients with glioblastoma in the subsequent period and in the United States. Given the considerable proportion of elderly glioblastoma patients, the worse outcome of the elderly population, and the paucity of comprehensive investigation of trends for this important subgroup, we aim to conduct this study to provide in-depth description of the patterns of incidence trends and to examine the age-period-cohort effects to the trends of glioblastoma specific to elderly patients.

## Methods

### Data sources and study cohort

Data were derived from the Surveillance Epidemiology and End Results (SEER) 18 registry database, which consists of cancer registries from 18 geographic areas, covering approximately 28% of the US population. In this study, patients aged over 65 years with a diagnosis of glioblastoma, according to the International Classification of Diseases for Oncology, Third Edition (ICD-O-3) histology codes 9440–9442 from 2000 to 2017 were included. Demographic and tumor characteristics of included patients were obtained. Demographic characteristics included gender, race, calendar year of diagnosis, and age at diagnosis. Race was categorized into non-Hispanic White, non-Hispanic Black, Hispanic, Asian/Pacific Islander (API), and American Indian/Alaskan Native (AIAN). Age at diagnosis was grouped as 65–69 years, 70–74 years, 75–79 years, 80–84 years, and 85+ years. Tumor characteristics included ICD-O-3 histologic type, tumor location, and tumor size. Histologic types were classified as 9440/3-glioblastoma, NOS, 9441/3-giant cell glioblastoma, and 9442/3-gliosarcoma. The topography of tumor was divided into supratentorial (ICD-O-3 site codes C710-C714), infratentorial (C716-C717), and overlapping/NOS (C715, C718-C719). Tumor sizes were categorized into < 4 cm, ≥4 cm, and unknown, according to Collaborative Stage Tumor Size. However, documentation of tumor size was only accessible for records between 2004 and 2015.

### Statistical analysis

Incidence rate, incidence rate ratio (IRR) and other relevant statistics were calculated using SEER*Stat (version 8.3.8, https://seer.cancer.gov/seerstat/). Figures were generated using R software (version 3.6.3). The incidence rates were age-adjusted and standardized to the 2000 US population, and reported per 100,000 population with 95% CI. The age-adjusted rate for an specific age group which comprised of the age x through y is calculated using the following formula (https://seer.cancer.gov/seerstat/tutorials/aarates/definition.html):

$$ Age\ {adjusted\ rate}_{x-y}=\sum \limits_{i=x}^y\left[\left(\frac{count_i}{population_i}\right)\times \mathrm{100,000}\times \left(\frac{{standard\ population}_i}{\sum_{j=x}^y{standard\ population}_j}\right)\right] $$

Joinpoint Regression Program software (version 4.8.0.1, https://surveillance.cancer.gov/joinpoint/) was used to calculate APC to quantify the trends of incidence across the years studied with the simplest join-point model. Joinpoint Regression Program establishes models with minimum to maximum number, which are supplied by the user, of join-points and uses the Monte Carlo Permutation method to test whether an apparent change in trends is statistically significant [10]. In this study, the minimum number and maximum number of join-points were set to 0 and 3, respectively. APC by gender, race, age at diagnosis, histological type, tumor location, and tumor size was analyzed. In order to ensure that the statistical analyses were sufficiently stable, group-specific data with a sample size less than 50 were excluded from the analysis.

Age-period-cohort models were used to investigate the effects of age, period (year of diagnosis), and cohort (year of birth) on the change trends of glioblastoma incidence. The model was fitted to the crude incidence rates using the age-period-cohort analysis web tool developed by Rosenberg et al. [11]. Age was grouped into 11 two-year intervals (65–66, 67–68, … 83–84, 85+ years). Year of diagnosis was grouped into 9 two-year categories (2000–2001, 2002–2003 … 2014–2015, 2016–2017). Cohort was indexed by midyear of birth (1915, 1917 … 1949, 1951). The reference age, period and cohort were defined as the median of each range. Five age-period-cohort parameters and functions were calculated and presented in the study, including net drift, local drift, longitudinal age curve, period rate ratios (RR), and cohort RR [11]. Net drift is the analog of estimated APC of incidence rates. Local drift represents the estimated APC over time specific to age group. Longitudinal age curve depicts the longitudinal age-specific and cohort-specific rates adjusted for period deviations, which is generally considered superior to a cross-sectional age curve in assessing age effects [12]. Period RR and cohort RR are ratios of age-specific rates in each calendar period relative to the reference period (herein 2008–2009) and in each birth cohort relative to the reference cohort (herein the 1933 cohort), respectively.

## Results

### Demographic and tumor characteristics

Of the 24,417 elderly patients in the SEER 18 registry database identified by the three specific ICD-O-3 codes from 2000 to 2017, non-Hispanic whites (20,406, 83.6%) made up the majority. The overall incidence rate of elderly patients with glioblastoma was 13.16 per 100,000 population (95% CI, 12.99–13.32) from 2000 to 2017. The incidence rate of male was about 1.62 times that of female (16.85 [95% CI, 16.57–17.14] vs 10.39 [95% CI, 10.20–10.59] per 100,000 population). Table 1 shows detailed patient characteristics and related incidence rates.

**Table 1:** Age-adjusted incidence of demographic and tumor characteristics of elderly patients with glioblastoma: The SEER 18 registry database, 2000–2017

Characteristics	No. of patients(%)	Age-adjusted incidence rate(95% CI)
**Overall**	24,417 (100)	13.16 (12.99–13.32)
**Gender**		
Male	13,456 (55.1)	16.85 (16.57–17.14)
Female	10,961 (44.9)	10.39 (10.20–10.59)
**Race**		
Non-Hispanic White	20,406 (83.6)	15.10 (14.89–15.31)
Non-Hispanic Black	1089 (4.5)	6.92 (6.51–7.35)
Hispanic	1900 (7.8)	10.58 (10.11–11.08)
API	927 (3.8)	5.90 (5.52–6.29)
AIAN	61 (0.3)	5.52 (4.20–7.14)
**Age at diagnosis, years**		
65–69	6738 (27.6)	11.67 (11.39–11.95)
70–74	6287 (25.7)	14.12 (13.77–14.47)
75–79	5412 (22.2)	15.47 (15.06–15.89)
80–84	3700 (15.2)	14.24 (13.78–14.70)
≥85	2280 (9.3)	9.19 (8.81–9.57)
**Histologic type**		
Glioblastoma, NOS	23,762 (97.3)	12.80 (12.64–12.97)
Giant cell glioblastoma	172 (0.7)	0.09 (0.08–0.11)
Gliosarcoma	483 (2.0)	0.26 (0.24–0.28)
**Tumor location**		
Supratentorial	18,089 (74.1)	9.74 (9.60–9.89)
Frontal	6220 (25.5)	3.34 (3.26–3.43)
Temporal	6059 (24.8)	3.27 (3.19–3.35)
Parietal	3911 (16.0)	2.11 (2.04–2.17)
Occipital	1148 (4.7)	0.62 (0.59–0.66)
Infratentorial	195 (0.8)	0.11 (0.09–0.12)
Overlapping/NOS	6133 (25.1)	3.31 (3.22–3.39)
**Tumor size, cm**^**a**^		
< 4	5082 (30.7)	2.73 (2.65–2.81)
≥4	8387 (50.7)	4.51 (4.42–4.61)
Unknown	3058 (18.5)	2.47 (2.38–2.56)

*Abbreviations: CI* confidence interval, *API* Asian/Pacific Islander, *AIAN* American Indian/Alaskan Native, *NOS* not otherwise specified

^a^ Data are only documented between 2004 and 2015

### Comparison of glioblastoma incidence between the elderly and the young

**Fig. 1:**
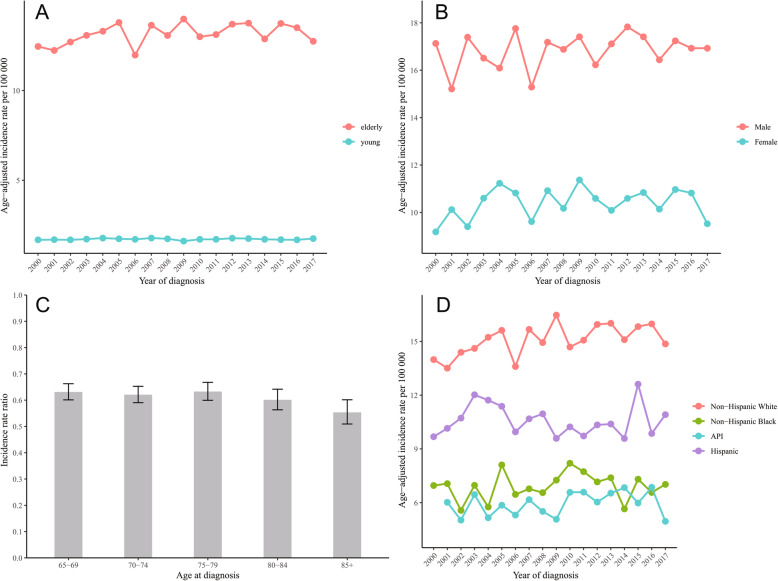
Annual age-adjusted incidence rates of glioblastoma (**a**) in the elderly and the young, and in the elderly by (**b**) gender, and (**d**) race. Incidence rate ratios of elderly glioblastoma for (**c**) gender by 5-year age groups

Figure 1a demonstrates the incidences of glioblastoma in the elderly (≥65 years) and in the young (< 65 years) from 2000 to 2017. The overall incidence of glioblastoma in the young was 1.76 per 100,000 population (95% CI, 1.70–1.74). The incidence trend of glioblastoma in the young remained stable between 2000 to 2017 (APC 0.0, 95% CI, − 0.2 to 0.3, *p* = 0.850). Compared with the young, the elder had approximately 8-fold the risk of glioblastoma. The incidence of glioblastoma in the elderly reached lowest in 2006 (Incidence rate 11.97 per 100,000 population, 95% CI, 11.28–12.69) and peaked in 2009 (Incidence rate 13.98 per 100,000 population, 95% CI, 13.25–14.73). The trend of incidence in the elderly also remained stable and there was a non-significant increasing tendency (APC 0.3, 95% CI, − 0.1 to 0.7, *p* = 0.111).

### Incidence trends of elderly glioblastoma by gender, race and age at diagnosis

Figure 1b shows the incidence of male and female patients aged over 65 years with glioblastoma across the 18-year study period. The trend of incidence remained stable for both male patients (APC 0.2, 95% CI, − 0.2 to 0.6, *p* = 0.272) and female patients (APC 0.2, 95% CI, − 0.4 to 0.8, *p* = 0.402) (Fig. 1b, Table 2). The IRRs of female to male patients stratified by age at diagnosis are displayed in Fig. 1c. Female to male IRR ranged from 0.55 to 0.63 and was lowest in 85+ years group (Fig. 1c).

**Table 2:** Trends of glioblastoma incidence in the elderly population: The SEER 18 registry database, 2000–201

Characteristics	Period	APC (95% CI)	P
**Overall**	2000–2017	0.3 (−0.1, 0.7)	0.111
**Gender**			
Male	2000–2017	0.2 (− 0.2, 0.6)	0.272
Female	2000–2017	0.2 (− 0.4, 0.8)	0.402
**Race**			
Non-Hispanic White	2000–2017	0.6 (0.2, 1.1)	0.013^c^
Non-Hispanic Black	2000–2017	0.2 (−0.8, 1.3)	0.648
Hispanic	2000–2017	0.0 (−0.9, 0.8)	0.923
API	2001–2017	0.7 (−0.6, 1.9)	0.266
**Age at diagnosis, years**			
65–69	2000–2017	0.3 (−0.2, 0.8)	0.219
70–74	2000–2017	0.0 (−0.6, 0.6)	0.976
75–79	2000–2017	0.3 (−0.5, 1.0)	0.482
80–84	2000–2017	0.8 (−0.1, 1.7)	0.092
≥85	2000–2017	0.6 (−0.3, 1.4)	0.186
**Histologic type**			
Glioblastoma, NOS	2000–2017	0.3 (−0.1, 0.7)	0.107
Giant cell glioblastoma	2000–2017	−2.3 (−6.1, 1.7)	0.239
Gliosarcoma	2000–2017	1.2 (−1.0, 3.4)	0.264
**Tumor location**			
Supratentorial	2000–2017	0.7 (0.2, 1.3)	0.016^c^
Supratentorial^a^	2000–2004	5.1 (0.3, 10.2)	0.039^c^
Supratentorial^a^	2004–2017	0.0 (−0.6, 0.7)	0.899
Frontal	2000–2017	1.7 (1.0, 2.5)	< 0.001^c^
Temporal	2000–2017	0.9 (0.3, 1.6)	0.010^c^
Parietal	2000–2017	−0.7 (−1.3, 0.0)	0.060
Occipital	2000–2017	−0.4 (−1.7, 0.9)	0.494
Infratentorial	2000–2017	−0.4 (−3.3, 2.5)	0.750
Overlapping/NOS	2000–2017	−0.9 (−1.6, − 0.2)	0.012^c^
**Tumor size, cm**^**b**^			
< 4	2004–2015	2.5 (1.4, 3.6)	< 0.001^c^
≥4	2004–2015	0.8 (0.0, 1.6)	0.057
Unknown	2004–2015	−4.9 (−6.6, −3.3)	< 0.001^c^

*Abbreviations: APC* annual percentage change, *CI* confidence interval, *API* Asian/Pacific Islander, *NOS* not otherwise specified

^a^ A statistically significant change in the APC was observed after join-point analysis

^b^ Data are only documented between 2004 and 2015

^c^ Statistically significant

Incidence rates of non-Hispanic white patients were higher than that of other races across all years and increased significantly by an average of 0.6% (95% CI, 0.2 to 1.1%, *p* = 0.01) annually during the years investigated (Fig. 1d, Table 2). There were no significant changes in incidence rates of non-Hispanic blacks, Hispanic population and API population (APC 0.2, 95% CI, − 0.8 to 1.3, *p* = 0.648; APC 0.0, 95%CI, − 0.9 to 0.8, *p* = 0.923; APC 0.7, 95% CI, − 0.6 to 1.9, *p* = 0.266, respectively) (Fig. 1d, Table 2). APC of AIAN population was not calculated and the incidence trend is not shown in Fig. 1d due to insufficient sample size.

**Fig. 2:**
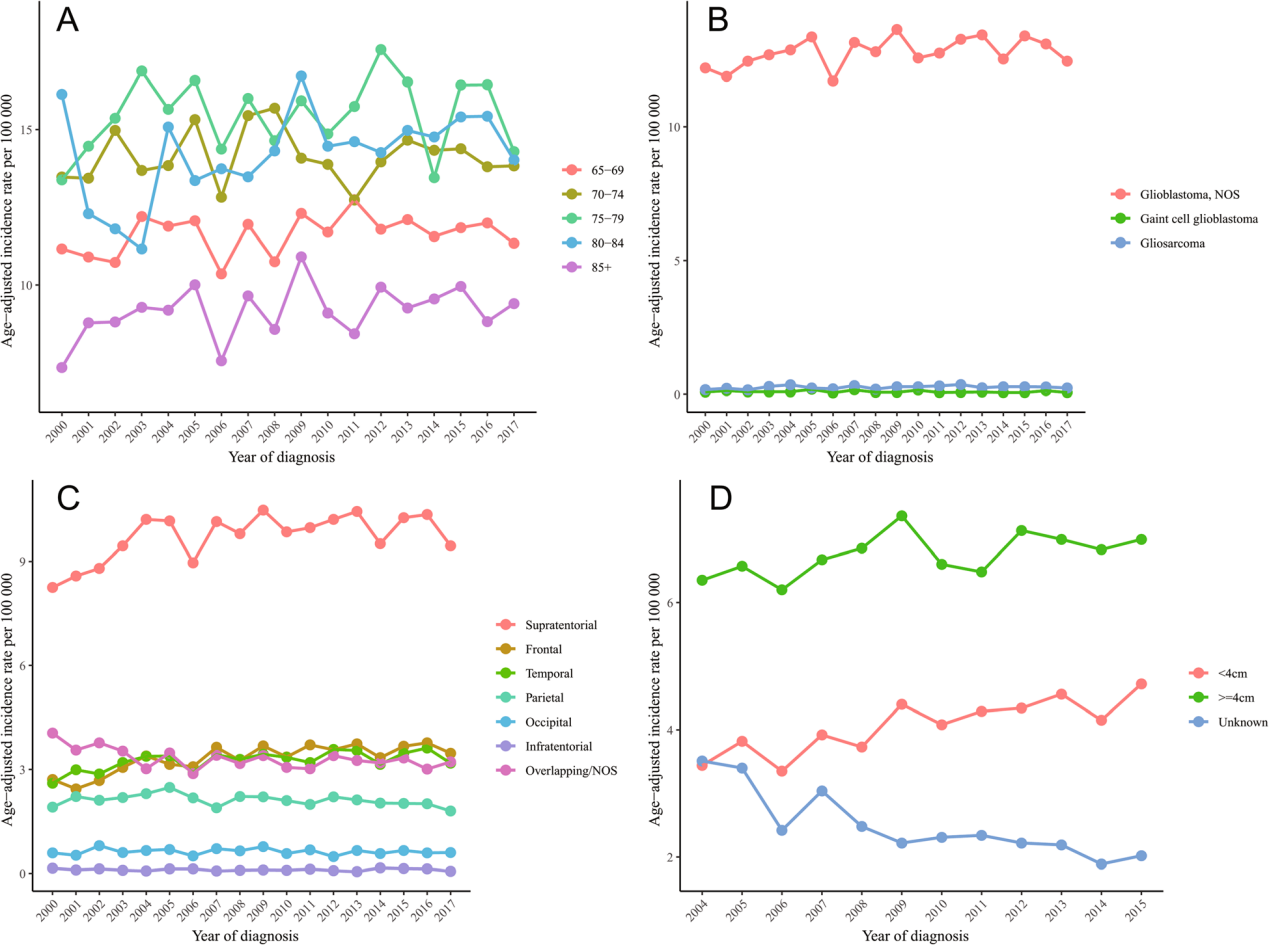
Annual age-adjusted incidence rates for elderly glioblastoma by (**a**) 5-year age groups, (**b**) histological type, (**c**) location, and (**d**) tumor size

Figure 2a showed that the incidence rates did not change significantly in all the 5-year age groups (65–69 years, APC 0.3, 95% CI, − 0.2 to 0.8, *p* = 0.219; 70–74 years, APC 0, 95% CI, − 0.6 to 0.6, *p* = 0.976; 75–79 years, APC 0.3, 95% CI, − 0.5 to 1.0, *p* = 0.482; 80–84 years, APC 0.8, 95% CI, − 0.1 to 1.7, *p* = 0.092; 85+ years, APC 0.6, 95% CI, − 0.3 to 1.5, *p* = 0.186) (Table 2).

### Incidence trends of elderly glioblastoma by histological type, tumor location and size

There was a non-significantly increasing incidence trend of glioblastoma, NOS, which was the most common histological type (APC 0.3, 95% CI, − 0.1 to 0.7, *p* = 0.107) (Fig. 2b, Table 2). The absolute change in incidence rate of giant cell glioblastoma and gliosarcoma was moderate due to relatively low incidence (Fig. 2b, Table 2).

The incidence rates of supratentorial glioblastoma increased significantly from 2000 to 2004 (APC 5.1, 95% CI, 0.3 to 10.2, *p* = 0.039) and remained stable from 2004 to 2017 (APC 0, 95% CI, − 0.6 to 0.7, *p* = 0.899), resulting in a significantly increasing trend of incidence across the years studied (APC 0.7, 95% CI, 0.2 to 1.3, *p* = 0.016) (Fig. 2c, Table 2). APC of incidence for infratentorial glioblastoma was − 0.4 (95% CI, − 3.3 to 2.5, *p* = 0.750) (Fig. 2c, Table 2). A significantly decreasing trend of incidence was observed for overlapping/NOS glioblastoma (APC -0.9, 95% CI, − 1.6 to − 0.2, *p* = 0.012) (Fig. 2c, Table 2). Among supratentorial glioblastoma, incidences increased significantly for tumors located at frontal lobe and temporal lobe (APC 1.7, 95% CI, 1.0 to 2.5, *p* < 0.001; APC 0.9, 95% CI, 0.3 to 1.6, *p* = 0.010, respectively) (Fig. 2c, Table 2). There is a non-significantly decreasing tendency for tumors located at parietal lobe and occipital lobe (APC -0.7, 95%CI, − 1.3 to 0, *p* = 0.060; APC -0.4, 95% CI, − 1.7 to 0.9, *p* = 0.494, respectively) (Fig. 2c, Table 2).

When stratifying the incidence of elderly glioblastoma by tumor size, incidence of cases with a tumor smaller than 4 cm increased significantly from 3.44 per 100,000 population (95% CI, 3.07–3.84) in 2004 to 4.72 per 100,000 population (95% CI, 4.34–5.13) in 2015 (APC 2.5, 95% CI, 1.4 to 3.6, p < 0.001) (Fig. 2d, Table 2). There was a non-significant increasing tendency for glioblastomas ≥ 4 cm (APC 0.8, 95 CI, 0 to 1.6, *p* = 0.057) (Fig. 2d, Table 2). The incidence of glioblastomas with unknown tumor sizes, however, decreased significantly from 2004 to 2015 (APC -4.9, 95% CI, − 6.6 to − 3.3, p < 0.001) (Fig. 2d, Table 2).

### Age-period-cohort analysis

**Fig. 3:**
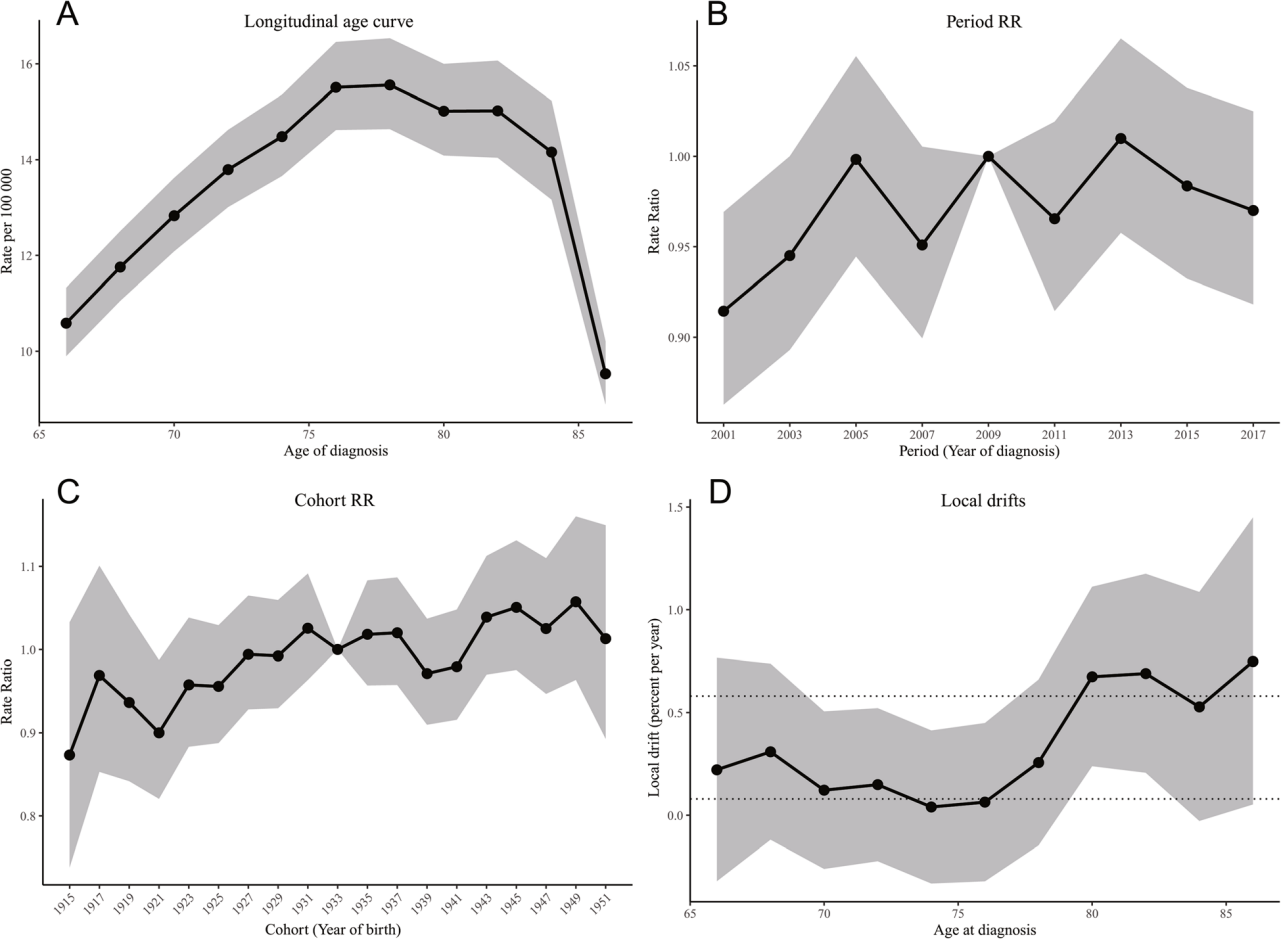
Age-period-cohort parameters and functions for the incidence of glioblastoma in the elderly, including (**a**) longitudinal age curve, (**b**) period RR, (**c**) cohort RR, and (**d**) local drifts with net drift. The shaded gray regions represent the 95% confidence interval. The solid and dot horizontal lines in (**d**) represent net drift and the 95% confidence interval. RR, rate ratio

The age-period-cohort modelling showed the effect of age on incidence trends (*p*< 0.001, Wald test), while did not indicate the period and cohort effects of the incidence trends of glioblastoma (*p* = 0.063 and *p* =0.536, respectively, Wald test). The longitudinal age curve is displayed in Fig. 3a. The risk of glioblastoma increased with age from 65 to 66 years (10.589 per 100,000, 95% CI, 9.9–11.326) to 77–78 years (15.559 per 100,000, 95% CI, 14.46–16.535), and for patients older than 78 years, the risk decreased with age and was lowest in patients aged over 85 years (9.528 per 100,000, 95% CI, 8.892–10.21). The period and cohort effects on glioblastoma are displayed in Fig. 3b and Fig. 3c. The relative risks remained relatively stable for both periods and birth cohorts. Figure 3d demonstrates the local drift values, which is the analog of the age-specific APC within the period studied. The overall net drift of 0.3% per year (95% CI, 0.1–0.6%) was quite modest. The local drifts were all above 0 and were higher with advanced age, which peaked at 0.7% per year (95% CI, 0.1–1.5%) in patients aged over 85 years.

## Discussion

This review of the SEER 18 registry database with a 18-year study period found that the age-adjusted incidence of glioblastoma in the elderly was 13.16 per 100,000 population, which is approximately 4 times higher than the overall incidence of glioblastoma according to the latest CBTRUS statistical report [1]. In contrast with previous work reporting an increasing trend of elderly glioblastoma, the overall age-adjusted incidence of glioblastoma in the elderly population was stable in recent years according to this study [8, 9]. This discrepancy is likely due to shorter study periods in previous reports along with dissimilar ancestry of populations. In addition, we found the incidence of most subgroups did not change significantly except for non-Hispanic white population, supratentorial location (mostly attributed to frontal and temporal lobes), smaller tumor size (< 4 cm), which increased significantly, and overlapping/NOS location, unknown tumor size, which declined significantly. The age-period-cohort analysis showed age effects on the trend of glioblastoma while did not show period and cohort effects on the incidence trend.

The incidence of glioblastoma varies in different age groups. Unlike meningioma, whose incidence tended to persistently increase in the oldest age group, the incidence of glioblastoma peaked in the age group of 75 to 79 years and then declined in 80–84 and 85+ years age groups [13]. This was also validated in the age-period-cohort analysis. Global life expectancy was forecasted to potentially increase to 77.8 years for males and 82.5 years for females in 2040 [14]. With the growing and aging population, glioblastoma, primarily a disease of the elderly with poor prognosis, should set alarm bells ringing.

Males were more frequently affected than females by most histological types of primary brain or other central nervous system tumors, including astrocytoma, oligodendroglioma, oligoastrocytic tumors, ependymal tumors, embryonal tumors, lymphoma, and germ cell tumors [1]. This study reported a glioblastoma Male: Female IRR of 1.62 among elderly patients, which is in consistence with previous work. We also identified a significant male predominance for every five-year age group. The sexual difference of glioblastoma incidence cannot be solely explained by the effect of acute sex hormone action due to the similar distribution among different age groups [15, 16]. Molecular subtype-specific effects of incidence were reported in different subtypes of glioblastoma [16–19]. The Mesenchymal subtype with a combination of loss of NF1, PTEN, and TP53 function had the greatest Male: Female IRR of about 2:1 [18]. While the Classical subtype with EGFR mutation/amplification and CDKN2A deletion occurred equally in males and females [16]. However, due to the limitations for SEER-derived data, this study was unable to evaluate the difference in incidence by molecular subgroup. Further studies exploring sex differences in molecular mechanisms are required. Additionally, fetal microchimerism, which develops from the fetal cells transferred through the placenta to mothers during pregnancy and may remain a lifelong time, was found in approximately 80% female glioblastoma cases [20]. However, no specific clinical or molecular characteristics of glioblastoma was identified to associate with fetal microchimerism [20].

Like other gliomas, the incidence of glioblastoma was higher in non-Hispanic white population than any other race [21]. Glioblastoma incidence was also highest in regions with majority white populations, including North Europe and Canada [22]. Of noting, non-Hispanic white population was the only race subgroup that had an increasing trend of glioblastoma incidence. The root of this disparity may be partly attributed to the variance of hereditary susceptibility between races. A genome-wide association study suggested that increased European ancestry in non-European population might be associated with the occurrence of glioma and identified four novel susceptibility variants, including 7q21.11 (SEMA3A), 11p11.12 (Intergenic), 12q24.21 (RBM19) and 20p12.13 (HAO1, BMP2) [23]. Researchers have identified specific hereditary syndromes, such as Lynch and Li-Fraumeni, that are risk factors of glioma, although these diseases are rare [24]. This disparity may also partly due to the unequal chance of access to high-column hospitals for different races, thus non-whites which had worse access compared with whites might be misdiagnosed or underdiagnosed more frequently [25].

Most glioblastomas located above the tentorium cerebelli. Among these tumors, they were found to occur more commonly in frontal lobe and temporal lobe. There was an increasing incidence trend of supratentorial glioblastoma, which was mostly attributed to the two lobes mentioned above. It was reported that isocitrate dehydrogenase (IDH) mutant glioblastoma, which represents a minority of patients with glioblastoma (approximately 10%) and is more commonly diagnosed in younger patients, preferentially locates at frontal lobe [26, 27]. There was possibility that the increased incidence of frontal glioblastoma was partly attributed to the changes of molecular subtypes among elderly population. However, as data of IDH mutation are not documented in SEER database, we are unable to explore the incidence trend of IDH-wild and IDH-mutant glioblastoma. Further research into this hypothesis is warranted.

We also identified an increasing glioblastoma incidence trend of smaller tumor size (< 4 cm) in the elderly population. This might be attributed to the advances in neuroimaging which facilitates early detection. However, this cannot explain the relatively stable overall glioblastoma incidence. Additionally, the quality of collection and record of cases in SEER registry database has improved, resulting in a decreasing incidence trend of unknown tumor size.

Period and cohort effects are interpreted as risk factors or changes of diagnostic criteria or disease classification that may affect all ages equally or differently [12, 28, 29]. The age-period-cohort analysis of this study did not show period and cohort effects on the incidence trend of glioblastoma. Accumulating evidence have suggested that environmental factors are thought to be responsible for the occurrence of tumors [30]. However, there are only few validated environmental risk factors for glioblastoma. Ionizing radiation exposure is the only and modifiable environmental factor that facilitates glioblastoma development [31, 32]. A retrospective cohort study found a positive association between exposure to computed tomography and glioma with an excessive relative risk per mGy of 0.019 (*p* = 0.0033), although it recruited only children and the overall incidence remained low [33]. The association between usage of cell phone and glioma has caught attention. However, various reports have drawn inconsistent conclusions regarding the relationship between cell phone exposure and the development of glioma [34–37]. However, the result of age-period-cohort analysis may suggest that these factors had little effect on the incidence of glioblastoma in the elderly.

Davis et al. addressed several data-driven factors such as data collection practices and changes of WHO classification that have influenced the patterns of incidence trends of glioblastoma in England, Canada and the United States [38]. Misclassification of tumors by histology is less likely to happen in recent years due to improved accuracy of histological category and standardized data collection procedure in the United States. The stable incidence trend of elderly glioblastoma in this study can somehow reflect that data-driven factors have little effect on the incidence in recent years and in the United States.

There are some limitations that should be acknowledged. First, our analysis was based on the SEER 18 registry database, which only covers approximately 28% of the US population. The results of this study cannot represent the whole US population, despite high-quality collection and record of cases. Second, as discussed above, the molecular subtypes of glioblastoma are not specified in SEER database. The good news is that molecular information has been collected by the Central Cancer Registry in the United States since January 1, 2018 [1]. Future studies focusing on the incidence trends of specific molecular subtypes will further our understanding of glioblastoma. Third, data of the glioblastoma incidence were retrospectively collected, thus we were only able to comprehensively describe the trend of incidence. The underlying factors that may contribute to the trend were interpreted on speculation.

## Conclusion

In summary, the overall incidence of glioblastoma in the elderly population remained stable between 2000 and 2017. Male, non-Hispanic white predominance was observed, in accordance with previous reports. There was an increasing incidence trend of glioblastoma for non-Hispanic white race, supratentorial location, and smaller tumor size (< 4 cm) and a decreasing trend for overlapping/NOS location and unknown tumor size. Period and cohort effects were not evident in the trend of glioblastoma incidence. Future population-based studies exploring the difference in the trend of glioblastoma incidence by specific molecular subgroups are warranted to further our understanding of the etiology of glioblastoma.

## Data Availability

Data analyzed in this study was derived from SEER database (https://seer.cancer.gov/).

## Abbreviations

APC: Annual percentage change
CBTRUS: Central Brain Tumor Registry of the United States
SEER: Surveillance Epidemiology and End Results
ICD-O-3: International Classification of Diseases for Oncology, Third Edition
API: Asian/Pacific Islander
AIAN: American Indian/Alaskan Native
IRR: Incidence rate ratio
NOS: Not otherwise specified
IDH: Isocitrate dehydrogenase
